# Role of Far Infra-Red Therapy in Dialysis Arterio-Venous Fistula Maturation and Survival: Systematic Review and Meta-Analysis

**DOI:** 10.1371/journal.pone.0104931

**Published:** 2014-08-12

**Authors:** Khalid Bashar, Donagh Healy, Leonard D. Browne, Elrasheid A. H. Kheirelseid, Michael T. Walsh, Mary Clarke –. Moloney, Paul E. Burke, Eamon G. Kavanagh, Stewart Redmond Walsh

**Affiliations:** 1 Department of vascular surgery, University Hospital Limerick, Limerick, Ireland; 2 Centre for Applied Biomedical Engineering Research (CABER), Department of Mechanical, Aeronautical & Biomedical Engineering, Materials and Surface Science Institute, University of Limerick, Limerick, Ireland; 3 Department of surgery, National University of Ireland, Galway, Ireland; Medical University of Graz, Austria

## Abstract

**Introduction:**

A well-functioning arteriovenous fistula (AVF) is the best modality for vascular access in patients with end-stage renal disease (ESRD) requiring haemodialysis (HD). However, AVFs’ main disadvantage is the high rate of maturation failure, with approximately one third (20%–50%) not maturing into useful access. This review examine the use of Far-Infra Red therapy in an attempt to enhance both primary (unassisted) and secondary (assisted) patency rates for AVF in dialysis and pre-dialysis patients.

**Method:**

We performed an online search for observational studies and randomised controlled trials (RCTs) that evaluated FIR in patients with AVF. Eligible studies compared FIR with control treatment and reported at least one outcome measure relating to access survival. Primary patency and secondary patency rates were the main outcomes of interest.

**Results:**

Four RCTs (666 patients) were included. Unassisted patency assessed in 610 patients, and was significantly better among those who received FIR (228/311) compared to (185/299) controls (pooled risk ratio of 1.23 [1.12–1.35], p = 0.00001). In addition, the two studies which reported secondary patency rates showed significant difference in favour of FIR therapy- 160/168 patients - compared to 140/163 controls (pooled risk ratio of 1.11 [1.04–1.19], p = 0.003).

**Conclusion:**

FIR therapy may positively influence the complex process of AVF maturation improving both primary and secondary patency rates. However blinded RCTs performed by investigators with no commercial ties to FIR therapy technologies are needed.

## Introduction

The number of patients with end stage renal disease (ESRD) requiring haemodialysis (HD) is steadily rising, a trend that is expected to continue [1]. Vascular access is a critical component in successful HD. A well-functioning arteriovenous fistula (AVF) is the best modality for HD vascular access [2]–[6]. AVF maturation is a complex process of remodelling. The newly formed fistula has to form a low resistance circuit capable of dilation to accommodate the increased blood flow required for HD. The AVF also has to be cannulated repeatedly with ease. The need for re-intervention to maintain patency should be minimal [2]–[4], [6], [7].

AVFs’ main disadvantage is the high rate of maturation failure, with approximately one third (20%–50%) not maturing into useful access [8]–[10]. AVFs have higher primary failure rates to mature compared to grafts [8], [11], [12]. However they last longer, and with exclusion of fistulas that fail to mature primarily, the cumulative patency from formation to permanent failure is superior to grafts. AVFs also require fewer secondary interventions in the form of angioplasty, stenting or thrombectomy [8], [13]–[16]. AVFs are associated with fewer complications compared to AVG and CVC in terms of infection, death, vascular access salvage procedures and hospitalizations [15], [16]. Also, a mature AVF has a lower incidence of thrombosis and stenosis. This translates into prolonged patency rates and lower risk for infection [1], [17]–[20].

Maturation of AVF depends on variable biomechanical forces. Remodelling of the arterial limb is characterised by vessel dilatation and outward hypertrophic remodelling of the intimal layer. Remodelling at the venous end can be accompanied by aggressive intimal thickening resulting in inward hypertrophic remodelling. Intimal hyperplasia (IH) is defined as the abnormal migration and proliferation of vascular smooth muscle cells provoked by injury, inflammation or stretch with associated deposition of extracellular matrix in the intimal layer of the vein [21]–[23].

Far infra-red FIR therapy, which is a form of heat therapy, has been implicated in improvement of endothelial function and haemodynamics in coronary arteries, probably through up-regulating endothelial nitric oxide synthase (eNOS) expression in arterial endothelium leading to improved cardiac function in patients with chronic heart diseases [24]. Repeated leg hyperthermia using FIR has been shown to reduce oxidative stress in bed ridden type II diabetics [25].

FIR has also been reported to show encouraging results in phantom limb pain control [26], stimulation of the secretion of TGF-beta1 and activation of fibroblasts which may promote better wound healing independent of skin blood flow and skin temperature [27], [28], reduction of both stress and fatigue levels of patients with end stage renal disease (ESRD) and stimulates the autonomic nervous system in those who are receiving regular haemodialysis (HD) [29].

This review was designed to examine the effect of FIR on AVF maturation using primary and secondary patency rates as the main outcomes of interest.

## Methods

This systematic review and meta-analysis were conducted according to the Preferred Reporting Items for Systematic Review and Meta-Analysis (PRISMA) guidelines [30].

### Eligibility Criteria

We included observational studies or randomised controlled trials (RCTs) that examined FIR therapy in patients with AVFs and ESRD. Eligible studies reported on AVF patency rates in FIR and non-FIR groups at one year or more following initiation of FIR therapy. Cases series and case reports were excluded. There was no restriction with regard to publication status or language.

### Search strategy

A search of the literature for relevant studies was conducted in March 2014. We searched Medline without date restriction using the free text “far infra-red”. Additionally we used the strategy ([“far infra-red” OR “far infrared” OR “post conditioning”] AND [“arteriovenous fistula” “dialysis” OR “end stage renal disease” OR “dialysis access” OR “access survival” OR “primary patency” OR “secondary patency” OR “fistula maturation]”) to search CINAHL, EMBASE, the Cochrane library and Google Scholar. Bibliographies of included studies were searched for additional studies.

Abstracts of the relevant titles were subsequently obtained and evaluated for eligibility (KB, DH). Any remaining uncertainty was resolved by examination of the full article (KB, DH). Discussion with a third author (SRW) resolved discrepancies in cases of disagreement regarding eligibility.

The relevant outcomes for this review were primary patency – defined as unassisted AVF patency rates after at least 12 months of follow up - and secondary patency – defined as assisted patency rates after at least 12 months of follow up. The incidence of salvage procedures (endoluminal procedures or surgical procedures) for dysfunctional fistulas during follow up was a secondary outcome.

### Data Collection

Data were extracted and checked for accuracy by two reviewers (KB, DH) independently and recorded on a Microsoft Excel spreadsheet. Any disagreements in extracting data were discussed between two reviewers (KB, DH), and if not settled this was resolved by consulting with a third reviewer (SRW). The following information regarding participant characteristics were recorded: age, sex, presence of co-morbidities, start of HD, primary and secondary patency rates, AVF salvage procedures, underlying cause of ESRD, definition of first AVF malfunction and overall access survival. The trials’ inclusion and exclusion criteria were also recorded.

### Quality assessment for risk of bias

The risk of bias for each study was assessed according to the criteria outlined in the in the Cochrane Handbook for Systematic Reviews of Interventions [31]. For each included study; the method used to perform random sequence generation, allocation concealment and blinding was described. The study was then scrutinised for incomplete data outcomes, selective reporting and other potential sources of bias. Where possible, study protocols were obtained from trial registries to ascertain whether there was selective reporting within studies [Table 1].

**Table 1:** Results of the study quality assessment.

Included study	Domain	Support for judgement	DH’s judgement
Lai 2013 ESVS [37]	Random sequencegeneration	The method of generating the randomsequence was not described.	Unclear
	Allocationconcealment	No description of methods formaintaining allocation concealment.	Unclear
	Blinding ofparticipants andpersonnel	Participants and personnel were notblinded. Dysfunctional access signs andother referral criteria could have asubjective component.	High risk of bias
	Blinding of outcomeassessment	Outcome assessors were not blinded.Dysfunctional access signs and otherreferral criteria could have a subjectivecomponent.	High risk of bias
	Incomplete outcomedata	Loss to follow up was minimal.Analysis was not by intention to treat.9/59 control group patients crossed overto the intervention group potentiallyleading to bias in favour of theintervention	Unclear
	Selective reporting	No link to the protocol was given	Unclear
	Other sources of bias	None	Not available
Lin 2007J Am Soc Neph [35]	Random sequencegeneration	A computerised minimisation algorithmwas used	Low risk of bias
	Allocationconcealment	Allocation sequence was kept by a studynurse who would not disclose allocationsuntil time of intervention. Diagnosingmalfunction in a fistula could have had asubjective element.	Unclear
	Blinding ofparticipants andpersonnel	Participants and personnel were notblinded.	High risk of bias
	Blinding of outcomeassessment	Outcome assessors were not blinded.Diagnosing malfunction in a fistula couldhave had a subjective element.	High risk of bias
	Incomplete outcomedata	Loss to follow up was minimal and wassimilar between groups and was unlikelyto influence results	Low risk of bias
	Selective reporting	Protocol was not available.	Unclear
	Other sources of bias	None	Not available
Lin 2013 AJKD [38]	Random sequencegeneration	A computer generated sequence wasused	Low risk of bias
	Allocationconcealment	Sealed opaque envelope were used toconceal allocation. There was noinformation in the manuscript or protocolon who had access to the envelopes andwhether they were opened sequentially	Unclear
	Blinding ofparticipants andpersonnel	No blinding. Diagnosing malfunction in afistula could have had a subjective element.	High risk of bias
	Blinding of outcomeassessment	Ultrasonographers were blinded howeverpatients were not blinded. Diagnosingmalfunction in a fistula could have had asubjective element.	High risk of bias
	Incomplete outcome data	Loss to follow up was similar between groupsand unlikely to influence results	Low risk of bias
	Selective reporting	Link to protocol was provided(NCT01138254). Trial was notprospectively registered and there wereseveral changes made including changesto outcomes.	High risk of bias
	Other sources of bias	None	Not available
Lin 2013 Neph DialTransplant [36]	Random sequence generation	A computer generated sequence was used	Low risk of bias
	Allocationconcealment	Sealed opaque envelope were used toconceal allocation. Two study nurseshad access to the envelopes and therewas no information in the manuscript orprotocol on whether they were openedsequentially	Unclear
	Blinding ofparticipants andpersonnel	No blinding. Diagnosing malfunction in afistula could have had a subjective element.	High risk of bias
	Blinding of outcomeassessment	No blinding. Diagnosing malfunction in afistula could have had a subjective element.	High risk of bias
	Incomplete outcome data	Loss to follow up was similar betweengroups and unlikely to influence results	Low risk of bias.
	Selective reporting	All outcomes that were mentioned in theprotocol were reported. The subgroupingbased on polymorphisms of hemeoxygenase-1 was not prespecified	Low risk of bias
	Other sources of bias	None	Not available

### Data analysis

Statistical analyses were performed using Review Manager version 5.2.8 [32]. Pooled risk ratios were calculated using the random effects model of DerSimonian and Laird [33]. For continuous outcome variables the weighted mean difference (WMD) was calculated. The presence of statistical heterogeneity between studies was evaluated using the Cochran’s Q statistic. P-values less than 5% were considered as statistically significant. Publication bias was assessed visually using a funnel plot, and additionally by comparing fixed and random effects modelling in a sensitivity analysis – this is a recognised method that can detect the influence of small-study effects [34].

## Results

### Study Selection

**Figure 1:**
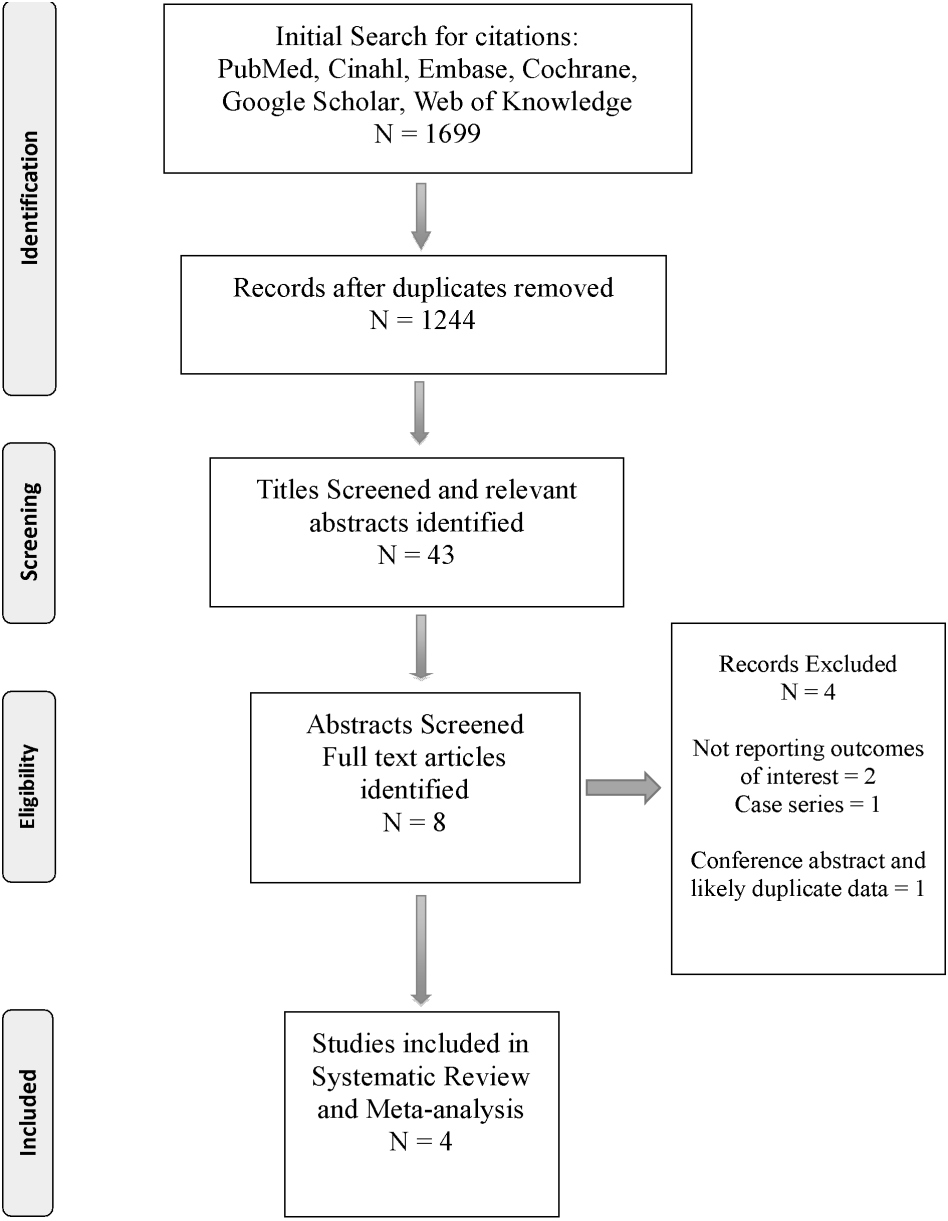
PRISMA 2009 Flow Diagram.

The results of the study selection process are summarized in the PRISMA flow diagram (Figure 1). The initial search yielded a total of 1669 citations, with 1244 citations remaining following removal of duplicates. The titles of these citations were screened with a total of 43 titles deemed potentially relevant. The abstracts of those titles were examined and eight full text articles were subsequently retrieved and examined. After assessing for eligibility criteria, four RCT’s were included in the review [35]–[38]. Three of those studies reported on patients with history of previous AVF who had been on HD prior to FIR therapy [35]–[37], while one study reported on patients with newly formed AVF not on HD [38]. We were not able to include another study by Lin et al [39] with a follow up of 3 months for primary patency rates as it was a conference abstract only, also we had concerns that the data in this study was used in another study by the same author [36] that has already been included in this review. Three studies were excluded from the final analysis after going through the full articles. Shipley et al followed their patients for six months in a case series of 20 patients – no control group - and reported maturation in 10 of those patients [40]. Two studies did not report on the outcomes of interest to the author of this systematic review [29], [41].

### Characteristics of included studies

#### Far Infrared (FIR) technique

All studies included used the same technique for delivering of FIR therapy. WS TY101 FIR emitter (WS Far Infrared Medical Technology Co., Ltd., Taipei, Taiwan) was used in all studies which generates electromagnetic waves with wavelengths in the range between 5 and 25 (peak at 5–8.2 µm). The top radiator was set at a height of 20–30 cm above the surface of the AVF with the treatment time set at 40 min during HD three times per week.

### Participants

The four studies included 666 patients, with 340 patients randomised to receive FIR therapy – median age 62.3±14.5 SD, while 326 were randomised to the control group – median age 62.0±14.5 SD. 348 patients were males–180 in FIR group and 168 in the control group, while females were 318, of those 160 received FIR therapy and 158 were controls.

Inclusion and exclusion criteria of studies are outlined in [Table 2], along with the definition of AVF malfunction for each of the included studies.

**Table 2:** Inclusion & exclusion criteria and definition of AVF malfunction for included studies.

Study	Inclusion criteria	Exclusion criteria	Definition of AVF malfunction
Lin 2007 [35]	(1) Receiving 4 h of maintenanceHD therapy three times weeklyfor at least 6 months at TaipeiVeterans General Hospital,(2) Using a native AVF as thecurrent vascular access for morethan 6 months, withoutinterventions within the last 3months, and (3) Creation ofAVF by cardiovascular surgeons inour hospital with the standardizedsurgical procedures of venousend-to-arterial side anastomosisin the upper extremity.	During the 1-yr follow-up,patients would be excludedfrom the study because of thefollowing censoring criterion:(1) Renal transplantation,(2) Death with a functioningaccess, (3) Shifting to peritonealdialysis, and (4) Loss offollow-up.	The need for any interventionalprocedure (surgery or angioplasty)to correct an occlusive ormalfunctioning AVF that cannotsustain an extracorporeal bloodflow >200 ml/min during HD afterexclusion of the following stenosis-unrelated events: Infectiouscomplication, progressiveaneurysmal formation, or stealsyndrome.
Lin 2013_AJKD [38]	(1) Aged 18–80 years, (2) HadCKD with estimated glomerularfiltration rate (eGFR) of 5–20 mL/min/1.73 m^2^, (3) Werenot anticipated to receive dialysisor kidney transplantation withinthe next 3 months, and (4) Wereundergoing AVF creation withvenous end-to-arterial sideanastomosis in the upperextremity.	(1) Those receiving anarteriovenous graft or cuffedtunnelled double-lumen catheteras the type of permanent vascularaccess, (2) Heart failure of NewYork Heart Association functionalclass III or IV, and (3) Episode ofcardio- or cerebrovascular event orreceiving intervention therapywithin 3 months prior to screening.	The need for any interventionalprocedure (surgery or angioplasty)to correct an occlusive ormalfunctioning fistula which couldnot sustain an extracorporeal bloodflow >200 mL/min during HD afterexcluding the following stenosis-unrelated events, such as infectiouscomplication, progressiveaneurysmalformation or steal syndrome.
Lai_2013 [37]	(1) Received two or more PTA onthe target lesions at upperextremities, with the last PTAsuccessfully performed within theweek before patient enrolment, and(2) After successful completion ofat least 1 week of HD treatment,the patients with AVF or AVGwere consecutively enrolled andrandomly assigned to either apost-PTA FIR radiation groupor a control group receiving theusual form radiation therapy at a1∶1 ratio.	(1) Received HD treatments otherthan three times a week, (2) Hadpreviously received FIR radiationTherapy, (3) Received implantationof an endovascular stent, (4) Hadmultiple lesions that a singleradiation field did not cover or thecentral lesion was considered toodeep to be irradiated, (5) MissedFIR radiation treatments exceeding10%, (6) Underwent renaltransplantation, (7) Switched toperitoneal dialysis treatments, and(8) Had any severe disease with anestimated life expectancy of lessthan 1 year.	A significant lesion was definedas a lumen loss of 50% or morecompared with adjacent normalvessel on angiography followingdysfunctional diagnosis based onclinical signs suggestive of stenosis.
Lin_2013_NDT [36]	(1) Receiving 4 h of maintenanceHD therapy three times weeklyfor at least 6 months at TaipeiVeterans General Hospital,(2) Using a native AVF as thepresent vascular access for >6months, without interventionswithin the last 3 months,and (3) Creation of AVF bycardiovascular surgeons in ourhospital with the standardizedsurgical procedures of venousend-to-arterial side anastomosisin the upper extremity.	Received an AV graft as the firstvascular access.	The need for any interventionalprocedure (surgery or angioplasty)to correct an occlusive ormalfunctioning fistula which couldnot sustain an extracorporeal bloodflow >200 mL/min during HD afterexcluding the following stenosis-unrelated events, such as infectiouscomplication, progressiveaneurysmal formation or stealsyndrome.

Apart from Lin et al who evaluated the effects of FIR in pre-dialysis patients with newly formed AVFs [38], the remaining RCTs included patients who already started HD. Mean time on HD in months for Lin et al [35] was 85.2±41.1 for FIR group and 79.2±42.2 for the control group, for Lai et al [37] FIR = 50.4±42 and control = 58.8±56.4 and for Lin et al in 2013 [36] FIR = 66.0±59.1 and control = 75.9±58.0. All patients in included trials received FIR therapy for 40 minutes per session three times a week for the duration of the study.

Lai et al studied patients with history of dysfunctional AVFs and repeated angioplasty. The mean life of the AVFs for their patients was 21.8±23.0 months for FIR group and 23.5±22.6 months for the controls [37]. 33 patients from 72 had history of AVF malfunction and 14 patients required surgical intervention, while 20 patients had a total of 49 angioplasty procedures in the FIR group compared to 34 patients from 73 with history of AVF malfunction, 13 of those required surgical intervention and 20 patients with total of 46 angioplasty procedures in the control group, in the RCT by Lin et al in 2007 [35]. Similarly, 47 patients had history of AVF malfunction with 12 patients requiring surgery from 139 and 35 patients underwent 79 angioplasty procedures in the FIR group, compared to 45 patients with history of malfunction, 13 patients of those required surgery and 32 patients underwent angioplasty as a salvage procedure in the control group in the study by Lin et al in 2013 [36]. All patients in both FIR and control groups had angioplasty procedures prior to recruitment in the study by Lai et al [37], while none of the patients included by Lin et al had a history of either surgical or angioplasty salvage procedures since they were all first time AVFs [38]. Lai et al had 9 of their patients who were initially randomised to the control group crossing over to the FIR group based on their request [37]. Clinical maturation was reported in 49 (81.7%) patients of 60 who received FIR therapy by Lin et all, compared to 37 (59.7%) from the 62 control subjects [38]. Sub –group analysis by age, gender and diagnosis of hypertension was not possible as this was not included in studies, and we did not have access to the raw data used by the authors. Other patients’ characteristics are detailed in [Table 3].

**Table 3:** Patients’ Characteristics across included studies.

Study	Patients		Age		Gender M:F		Diabetes		Hypertension		Hx of AVFfailure		Time on HD		Withdrawals
	FIR	Control	FIR	Control	FIR	Control	FIR	Control	FIR	Control	FIR	Control	FIR	Control	
Lin 2007[35]	63/72	64/73	61.9±14.4	59.2±19.0	37∶35	38∶35	25	24	40	39	33	34	85.2±41.1	79.2±42.2	Creation of another vascularaccess because of the poorresponse to angioplasty:1 patient receiving FIR therapyand four patients in control group.Patients were censored in case of:Renal transplantation (*n = *3), deathwith a functioning access (*n* = 5),shifting to peritoneal dialysis(*n* = 4) or loss of follow-up (*n = *1).
Lin2013_AJKD[38]	60	62	63.2±18.5	63.0±14.4	32∶28	35∶27	28	23	18	20	-	-	Pre-dialysis	Pre-dialysis	Lost to F/U: FIR = 1; Control = 1.Shifting to PD: FIR = 1; Control = 1.Death e AVF: FIR = 2; Control = 3.Renal transplantation: FIR = 1. NewAVF (Infection): Control = 1. D/Cintervention: FIR = 2; Control = 1.
Lin2013_NDT[36]	119/139	120/141	61.3±14.1	62.8±15.9	79∶60	71∶70	45	47	80	90	47	45	66.0±59.1	75.9±58.0	Underwent creation of anothervascular access due to non-stenoticlesions: 3 patients receiving FIRtherapy and 2 patients in thecontrol group. Patients werecensored in case of: Renaltransplantation (n = 5), death witha functioning access (n = 15), shiftto peritoneal dialysis (n = 5) orloss of follow-up (n = 9).
Lai 2013[37]	69	50	62.7±10.9	63.1±12.5	32∶37	24/26	42	28	48	38	All	All	50.4±42.0	58.8±56.4	Crossover patients: 9 fromControl to FIR.

### Primary - unassisted - patency rates at 1 year

**Figure 2:**
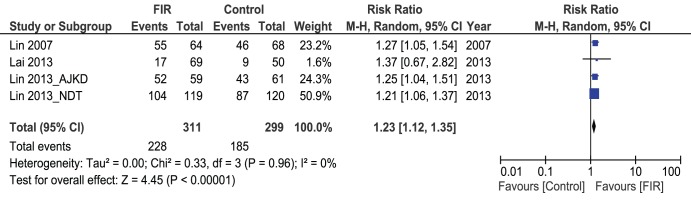
Forest Plot showing Primary AVFs patency at 12 months.

**Figure 3:**
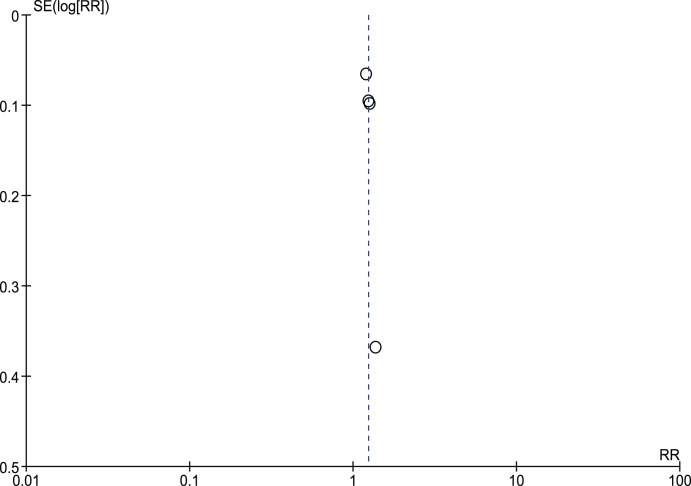
Funnel plot for Primary AVFs patency at 12 months.

All of the 4 included studies (610 patients) reported on unassisted – primary – patency rate after 12 months of follow-up on the FIR therapy. 228/311 patients in FIR group had primarily patent AVFs at 12 months compared to 185/299 patients in the control group. Pooled results showed significant difference between the two groups, with those who received FIR showing better primary patency rates compared to control (Pooled risk ratio = 1.23 [1.12, 1.35], 95% CI, p = 0.00001) [Figure 2]. There was no evidence of statistical heterogeneity (Cochran’s Q = 0.33; degree of freedom (DF) = 3; p = 0.96; I^2^ = 0%). The funnel plot did not suggest bias [Figure 3], and the result was unchanged when fixed effects modelling was used (pooled risk ratio1.24 [1.13–1.37], 95% CI, p<0.0001).

**Figure 4:**
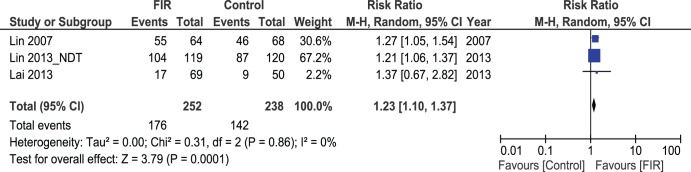
Forest Plot showing Primary AVFs patency at 12 months, Lin et al RCT on new AVFs excluded.

Excluding the RCT by Lin et al on newly formed AVFs in pre-dialysis patients [38] from the analysis for primary patency after 12 months, the remaining studies (490 patients) showed better results in the FIR group with 176/252 AVFs being patent at 12 months compared to 142/238 in the control group [35]–[37]. This was statistically significant (Pooled risk ratio = 1.23 [1.10, 1.37], 95% CI, p = 0.0001) [Figure 4]. There was no evidence of statistical heterogeneity (Cochran’s Q = 0.31; degree of freedom (DF) = 2; p = 0.86; I^2^ = 0%).

### Secondary - assisted - patency rates

**Figure 5:**
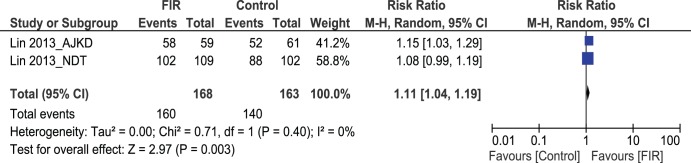
Forest plot showing assisted patency rates at 12 months.

Data could be retrieved from 2 studies (331 patients) for analysis of assisted – secondary – patency rates at 12 months following salvage procedures [36], [38]. 160/168 patients in the FIR group had patent AVFs following intervention for dysfunctional fistulas, compared to 140/163 patients in the control arm. Pooled results showed statistically significant difference favouring FIR therapy (Pooled risk ratio = 1.11 [1.04–1.19]; 95% CI, p = 0.003) [Figure 5]. There was no evidence of statistical heterogeneity (Cochran’s Q = 0.71; degree of freedom (DF) = 1; p = 0.40; I^2^ = 0%).

### Intervention

**Figure 6:**
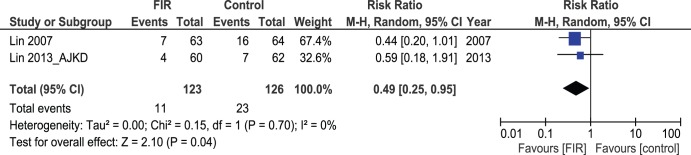
Forest plot showing surgical intervention for AVF malfunction.

Two studies [35], [38] (249 patients) reported the need for intervention to salvage a dysfunctional AVF. Patients who received FIR therapy required less interventions, 11/123 patients compared to 23/126 patients in the control group. The difference was significant (Pooled risk ratio = 0.49 [0.25–0.985; 95% CI; p = 0.04) [Figure 6]. There was no evidence of statistical heterogeneity (Cochran’s Q = 0.15; degree of freedom (DF) = 1; p = 0.70; I^2^ = 0%).

## Discussion

This review identified four studies (666 patients) which evaluated the use of FIR therapy to improve primary and secondary patency rates for AVFs in patients with ESRD. They all reported significant improvement in the outcome measures assessed in this review in favour of FIR therapy. Three of those studies (490 patients) were carried out on patients already started HD sessions, and one study (122 patients) focused on pre-dialysis first time AVF maturation. All four trials following some form of randomisation, and the demographics of patients in included studies did not differ significantly. Pooled analysis showed that primary - unassisted - patency was significantly better in the FIR group (pooled risk ratio of 1.23 [1.12–1.35], p value of 0.0001). Secondary – assisted – patency was reported in two studies (279 patients) and was found to be significantly better in those who received FIR therapy (pooled risk ratio of 1.19 [1.07–1.31], p value of 0.0008).

Post-conditioning using Far Infra-Red therapy has been shown to increase the level of heme oxygenase-1 (HO-1) expression which protects against Ischaemia/reperfusion injury in study by Tu et al [42]. HO-1 is a known vasodilator and at the same time inhibits proliferation of vascular smooth muscle cells, platelet aggregation, and vasospasm leading to favourable conditions for maturation of AVFs. Also, Ikeda et al repeated thermal therapy was shown to up-regulate endothelial nitric oxide synthase expression in Syrian hamsters [24], a finding that was validated by Akasaki et al, who also reported increased angiogenesis via (eNOS) following repeated thermal therapy in mice with hindlimb ischemia. [43]. Kipshidze et al irradiated cultures of rabbit endothelial cells and smooth muscle cells with different doses of non-ablative infrared. They found that non-ablative infrared laser inhibited neointimal hyperplasia after coronary arteries angioplasty in cholesterol-fed rabbits for up to 60 days [44]. FIR therapy is still considered a novel treatment for AVF although the technique has been described since 2007 by Lin et al [35]. This review demonstrated a beneficial use of FIR therapy that improved both primary and secondary patency rates across all studies included. This statistically significant difference was consistent even when one excluded study for having only 3 months of follow-up was added to the sensitivity analysis [39]. Also, excluding the only RCT found by the authors on newly formed AVF did not alter the outcome of the pooled analysis in terms of significance.

FIR therapy was also shown to improve access flow (Qa). The study by Lin et al – which was one of the RCTs included in the review - showed that 40 min of FIR therapy in a single HD session could increase access flow of AVF by about 50 mL/min with a 1-year effect of improving Qa by up to 150 mL/min and increasing unassisted patency of AVF by about 18% in comparison with controls [35].

A serious limiting factor of this systematic review is that the four RCTs came from the same institution (Yang-Ming University in Taiwan), and three of the four were authored by the same two authors (Lin-cc and Yang-wc) [35], [36], [38]. Dr Lin-cc reported that he was receiving lecture fees from WS Far Infrared Medical Technology, the company that makes the infrared machines used in the studies raising the potential of bias.

Also, all the RCTs were performed in an unblinded fashion, which can impact outcomes as demonstrated by the fact that in one study nine patients opted to join the FIR group despite initially being allocated as controls. Blinding in clinical trials involving FIR therapy would involve additional costs in making machines that resemble the ones used to deliver FIR therapy. Those machines should be convincing to both staff and patients if effective double blinding is to be considered. However, blinding can be attempted by placing a screen between the FIR device and the patient. Also, double-blinding can be achieved by placing a box over the device and then creating simple mock devices that also appear as boxes. This review provides a thorough examination of published evidence supporting the use of FIR therapy to promote AVF access maturation in patients with ESRD in HD, and also for those who are likely to require dialysis in the near future. The meta-analysis showed overwhelming support for regular use of FIR therapy, however there were limitations that need to be considered. Finally, this review may serve to guide future advances in using repeated thermal therapy in postconditioning of AVFs.

## Conclusion

Results from four RCTs suggest that regular use of FIR therapy in haemodialysis and pre-haemodialysis patients, in particular those with AVFs, can positively influence AVF function. However, more blinded randomised controlled, multicentre and international clinical trials are required. We also hope to see sub-group analysis in those studies, particularly by age (e.g. using 65 as cut-off), gender and diagnosis of hypertension.

## Supporting Information

Checklist S1 **PRISMA 2009 Checklist.**
(DOC)

## References

[1] Frankel A (2006) Temporary access and central venous catheters. Eur J Vasc Endovasc Surg 31: 417–422. 16360326 10.1016/j.ejvs.2005.10.003

[2] NKF-KDOQI (2006) 2006 Updates Clinical Practice Guidelines and Recommendations.

[3] Hoggard J, Saad T, Schon D, Vesely TM, Royer T (2008) Guidelines for venous access in patients with chronic kidney disease. A Position Statement from the American Society of Diagnostic and Interventional Nephrology, Clinical Practice Committee and the Association for Vascular Access. Semin Dial 21: 186–191. 18364015 10.1111/j.1525-139X.2008.00421.x

[4] Polkinghorne KR, Chin GK, Macginley RJ, Owen AR, Russell C, et al (2013) KHA-CARI guideline: Vascular access - central venous catheters, arteriovenous fistulae and arteriovenous grafts. Nephrology (Carlton). 10.1111/nep.12132 23855977

[5] Stolic R (2013) Most important chronic complications of arteriovenous fistulas for hemodialysis. Med Princ Pract 22: 220–228. 23128647 10.1159/000343669 PMC5586732

[6] McCann M, Einarsdottir H, Van Waeleghem JP, Murphy F, Sedgewick J (2008) Vascular access management 1: an overview. J Ren Care 34: 77–84. 18498572 10.1111/j.1755-6686.2008.00022.x

[7] Dixon BS (2006) Why don’t fistulas mature? Kidney Int 70: 1413–1422. 16883317 10.1038/sj.ki.5001747

[8] Allon M, Robbin ML (2002) Increasing arteriovenous fistulas in hemodialysis patients: problems and solutions. Kidney Int 62: 1109–1124. 12234281 10.1111/j.1523-1755.2002.kid551.x

[9] Rayner HC, Pisoni RL, Gillespie BW, Goodkin DA, Akiba T, et al (2003) Creation, cannulation and survival of arteriovenous fistulae: data from the Dialysis Outcomes and Practice Patterns Study. Kidney Int 63: 323–330. 12472799 10.1046/j.1523-1755.2003.00724.x

[10] Lok CE, Oliver MJ, Su J, Bhola C, Hannigan N, et al (2005) Arteriovenous fistula outcomes in the era of the elderly dialysis population. Kidney Int 67: 2462–2469. 15882293 10.1111/j.1523-1755.2005.00355.x

[11] Allon M, Lockhart ME, Lilly RZ, Gallichio MH, Young CJ, et al (2001) Effect of preoperative sonographic mapping on vascular access outcomes in hemodialysis patients. Kidney Int 60: 2013–2020. 11703621 10.1046/j.1523-1755.2001.00031.x

[12] Oliver MJ, McCann RL, Indridason OS, Butterly DW, Schwab SJ (2001) Comparison of transposed brachiobasilic fistulas to upper arm grafts and brachiocephalic fistulas. Kidney Int 60: 1532–1539. 11576369 10.1046/j.1523-1755.2001.00956.x

[13] Allon M (2007) Current management of vascular access. Clin J Am Soc Nephrol 2: 786–800. 17699495 10.2215/CJN.00860207

[14] Dixon BS, Novak L, Fangman J (2002) Hemodialysis vascular access survival: upper-arm native arteriovenous fistula. Am J Kidney Dis 39: 92–101. 11774107 10.1053/ajkd.2002.29886

[15] Ocak G, Rotmans JI, Vossen CY, Rosendaal FR, Krediet RT, et al (2013) Type of arteriovenous vascular access and association with patency and mortality. BMC Nephrol 14: 79. 23557085 10.1186/1471-2369-14-79 PMC3621613

[16] Polkinghorne KR, McDonald SP, Atkins RC, Kerr PG (2004) Vascular access and all-cause mortality: a propensity score analysis. J Am Soc Nephrol 15: 477–486. 14747396 10.1097/01.asn.0000109668.05157.05

[17] Spergel LM, Ravani P, Roy-Chaudhury P, Asif A, Besarab A (2007) Surgical salvage of the autogenous arteriovenous fistula (AVF). J Nephrol 20: 388–398. 17879203

[18] Brunori G, Bandera A, Valente F, Laudon A (2008) [Vascular access for dialysis in elderly: AVF versus permanent CVC]. G Ital Nefrol 25: 614–618. 19048553

[19] McCann M, Einarsdottir H, Van Waeleghem JP, Murphy F, Sedgewick J (2010) Vascular access management III: central venous catheters. J Ren Care 36: 25–33. 20214706 10.1111/j.1755-6686.2010.00138.x

[20] Ma A, Shroff R, Hothi D, Lopez MM, Veligratli F, et al (2013) A comparison of arteriovenous fistulas and central venous lines for long-term chronic haemodialysis. Pediatr Nephrol 28: 321–326. 23052655 10.1007/s00467-012-2318-2

[21] Cox JL, Chiasson DA, Gotlieb AI (1991) Stranger in a strange land: the pathogenesis of saphenous vein graft stenosis with emphasis on structural and functional differences between veins and arteries. Prog Cardiovasc Dis 34: 45–68. 2063013 10.1016/0033-0620(91)90019-i

[22] Davies MG, Hagen PO (1994) Pathobiology of intimal hyperplasia. Br J Surg 81: 1254–1269. 7953384 10.1002/bjs.1800810904

[23] Newby AC, Zaltsman AB (2000) Molecular mechanisms in intimal hyperplasia. J Pathol 190: 300–309. 10685064 10.1002/(SICI)1096-9896(200002)190:3<300::AID-PATH596>3.0.CO;2-I

[24] Ikeda Y, Biro S, Kamogawa Y, Yoshifuku S, Eto H, et al (2001) Repeated thermal therapy upregulates arterial endothelial nitric oxide synthase expression in Syrian golden hamsters. Jpn Circ J 65: 434–438. 11348049 10.1253/jcj.65.434

[25] Kawaura A, Tanida N, Kamitani M, Akiyama J, Mizutani M, et al (2010) The effect of leg hyperthermia using far infrared rays in bedridden subjects with type 2 diabetes mellitus. Acta Med Okayama 64: 143–147. 20424670 10.18926/AMO/32849

[26] Huang CY, Yang RS, Kuo TS, Hsu KH (2009) Phantom limb pain treated by far infrared ray. Conf Proc IEEE Eng Med Biol Soc 2009: 1589–1591. 19964539 10.1109/IEMBS.2009.5334124

[27] Toyokawa H, Matsui Y, Uhara J, Tsuchiya H, Teshima S, et al (2003) Promotive effects of far-infrared ray on full-thickness skin wound healing in rats. Exp Biol Med (Maywood) 228: 724–729. 12773705 10.1177/153537020322800612

[28] Capon A, Mordon S (2003) Can thermal lasers promote skin wound healing? Am J Clin Dermatol 4: 1–12. 12477368 10.2165/00128071-200304010-00001

[29] Su LH, Wu KD, Lee LS, Wang H, Liu CF (2009) Effects of far infrared acupoint stimulation on autonomic activity and quality of life in hemodialysis patients. Am J Chin Med 37: 215–226. 19507267 10.1142/S0192415X09006783

[30] Liberati A, Altman DG, Tetzlaff J, Mulrow C, Gotzsche PC, et al (2009) The PRISMA statement for reporting systematic reviews and meta-analyses of studies that evaluate health care interventions: explanation and elaboration. PLoS Med 6: e1000100. 19621070 10.1371/journal.pmed.1000100 PMC2707010

[31] Higgins JP, Altman DG, Gotzsche PC, Juni P, Moher D, et al (2011) The Cochrane Collaboration’s tool for assessing risk of bias in randomised trials. BMJ 343: d5928. 22008217 10.1136/bmj.d5928 PMC3196245

[32] The Nordic Cochrane Centre TCc (2012) Review Manager (RevMan) [Computer Program]. Version 5.2.

[33] DerSimonian R, Laird NM (1983) Evaluating the Effect of Coaching on SAT Scores: A Meta-Analysis. Harvard Educational Review 53: 1–15.

[34] Sterne JAC EM, Moher D (2011) Chapter 10: Addressing reporting biases. Higgins JPT, Green S (editors) Cochrane Handbook for Systematic Reviews of Intervention Version 5.1.0.

[35] Lin CC, Chang CF, Lai MY, Chen TW, Lee PC, et al (2007) Far-infrared therapy: a novel treatment to improve access blood flow and unassisted patency of arteriovenous fistula in hemodialysis patients. J Am Soc Nephrol 18: 985–992. 17267744 10.1681/ASN.2006050534

[36] Lin CC, Chung MY, Yang WC, Lin SJ, Lee PC (2013) Length polymorphisms of heme oxygenase-1 determine the effect of far-infrared therapy on the function of arteriovenous fistula in hemodialysis patients: a novel physicogenomic study. Nephrol Dial Transplant 28: 1284–1293. 23345623 10.1093/ndt/gfs608

[37] Lai CC, Fang HC, Mar GY, Liou JC, Tseng CJ, et al (2013) Post-angioplasty far infrared radiation therapy improves 1-year angioplasty-free hemodialysis access patency of recurrent obstructive lesions. Eur J Vasc Endovasc Surg 46: 726–732. 24119468 10.1016/j.ejvs.2013.09.018

[38] Lin CC, Yang WC, Chen MC, Liu WS, Yang CY, et al (2013) Effect of Far Infrared Therapy on Arteriovenous Fistula Maturation: An Open-Label Randomized Controlled Trial. Am J Kidney Dis. 10.1053/j.ajkd.2013.01.015 23474008

[39] W.-C LC-CY (2012) Far infrared therapy improves arteriovenous fistula maturation. Nephrology Dialysis Transplantation Conference: 50th ERA-EDTA Congress Istanbul Turkey Conference Start: 20120524 Conference End: 20120527.

[40] Shipley T, Adam J, Sweeney D, Fenwick S, Mansy H, et al. (2012) Does far infrared therapy aid av fistula maturation and maintenance?. Nephrology Dialysis Transplantation Conference: 49th ERA-EDTA Congress Paris France Conference Start: 20120524 Conference End: 20120527 Conference Publication: (varpagings) 27 (ii261).

[41] Lin CH, Lee LS, Su LH, Huang TC, Liu CF (2011) Thermal therapy in dialysis patients - a randomized trial. Am J Chin Med 39: 839–851. 21905276 10.1142/S0192415X1100924X

[42] Tu YP, Chen SC, Liu YH, Chen CF, Hour TC (2013) Postconditioning with far-infrared irradiation increases heme oxygenase-1 expression and protects against ischemia/reperfusion injury in rat testis. Life Sci 92: 35–41. 23142244 10.1016/j.lfs.2012.10.019

[43] Akasaki Y, Miyata M, Eto H, Shirasawa T, Hamada N, et al (2006) Repeated thermal therapy up-regulates endothelial nitric oxide synthase and augments angiogenesis in a mouse model of hindlimb ischemia. Circ J 70: 463–470. 16565566 10.1253/circj.70.463

[44] Kipshidze N, Nikolaychik V, Muckerheidi M, Keelan MH, Chekanov V, et al (2001) Effect of short pulsed nonablative infrared laser irradiation on vascular cells in vitro and neointimal hyperplasia in a rabbit balloon injury model. Circulation 104: 1850–1855. 11591625 10.1161/hc3901.096101

